# Cancer care in China: A general review

**DOI:** 10.2349/biij.4.3.e39

**Published:** 2008-07-01

**Authors:** XJ Ma, C Lin, W Zhen

**Affiliations:** 1 Department of Radiation Therapy, Qingdao Tumor Hospital, Qingdao, China; 2 Department of Radiation Oncology, University of Nebraska Medical Center, Omaha Nebraska, United States

**Keywords:** Cancer care, China

## Abstract

This article is to provide a general overview of cancer in China including the statistics, most common cancers, their epidemiological characteristics and the treatments.

## CANCER STATISTICS IN CHINA

The most recent epidemiological data has shown that over 2.2 million new cancer cases (1.4 million in men, 0.8 million in women) are diagnosed in China each year and approximately 1.6 million of the cancers result in mortality. Over the last 20 years, the cancer-related mortality rate has risen by 30%, which constitutes 25-35% of all deaths. From 2000 to 2005 alone, the total number of new cases increased by 14.6% with the most common sites being lung, liver and stomach in men, and breast, lung and stomach in women primarily as a result of population growth and aging [1]. In addition, the rising rates of lung cancer incidence (in both sexes) and breast cancer mean that there will be much greater increases in the number of cases at these two sites (27% for lung cancer in men, 38% for lung and breast cancer in women). Cancer has become the number one cause of death in China [2].

As of the end of 2006, there are 104.2 million people who are 65 years or older, constituting 7.9% of the entire population in China. At present, China also has 20% of the world’s population older than 60 years old, which has surpassed 150 million. China has become one of the aging countries, with the fastest growth and the largest aging population in the world. This is one of the major reasons for rising cancer incidence in China.

In urban populations, the leading causes of death are as follows; cancer, cerebral vascular accidents, cardiovascular, pulmonary, injury and poisoning, gastrointestinal, endocrinological and metabolical, urological, and psychiatric disorders. Although over the last five decades the overall cancer incidence has continued to climb, this trend is not observed in all types of cancers. What used to be the most common cancers such as gastric, cervical, penile, oesophageal and nasopharyngeal cancers have declined in various degrees, whereas lung, breast, colon, and prostate cancer rates have increased significantly (Table 1). This kind of shift in cancer rates is more evident in more developed coastal cities, which likely is due to the changes in lifestyle and diet (Table 2).

**Table 1:** The Leading Sites of New Cancer Cases in China over the last 30 years.

1970s	1990s	2000s
Stomach	Stomach	Lung
Oesophagus	Liver	Liver
Liver	Lung	Stomach
Lung	Oesophagus	Oesophagus
Cervix	Colorectal	Colorectal

**Table 2:** The percentage of change in annual mortality rates among urban and rural areas.

Type of Cancer	Annual mortality Rate (%)	
	Urban	Rural
Cervix	-27.5	-17.3
Lung	+29.4	+47.7
Liver	+13.0	+17.1
Colorectal	+31.2	+17.5
Breast	+38.9	+39.4
Bladder	+23.5	+19.9

The top five most common malignancies in China are lung, liver, gastric, oesophageal and colorectal cancers. The mortality of lung cancer has increased every year. For example, in Beijing, lung cancer death constitutes one-quarter of all cancer-related mortalities. According to the Initiative of Promoting Healthy Living in China (Cancer 2006), the mortality rate of lung cancer in China may exceed one million by 2025.

The main reasons for such a high lung cancer mortality rate in China are the following:

- Lack of awareness of lung cancer
- Lack of effective screening tools for early detection. Approximately 80% of lung cancers in China are advanced stage disease at the diagnosis
- Lack of access to health care
- Lack of health insurance and resources
- Lack of scientifically sound comprehensive care. It is estimated that about one-third of lung cancer patients may have died from inadequate or inappropriate therapies in China.

## THE CAUSE AND AETIOLOGY FOR COMMON CANCERS IN CHINA

### Lung cancer

- Tobacco use and inhalation of second hand smoke have clearly become the main causes of lung cancer.
- Rapid growth of aging population
- Rapid industrialisation and westernisation of cities
- Rapid urbanisation of rural communities
- Worsening pollution, especially air and water pollution
- Unhealthy lifestyles

The risk factors of lung cancer in China:

Smoking: China is the largest producer of tobacco products in the world; 2.5 times more than that of the United States, which is the second largest producer. China is also the largest consumer of tobacco in the world. The smoker population in China is estimated to be over 350 million. As many as 540 million Chinese are exposed to second-hand smoke, of which 180 million are under the age of 15 years, according to a national tobacco control report released on May 29, 2007 by the Ministry of Health of China. Women and children are most vulnerable to second-hand smoke with the smoking rate among men reaching 57%. Ninety percent of the women are exposed to second-hand smoke at home. There has been no collective effort to curtail the use of tobacco products. In 2005, cigarette sales generated US$32.5 billion in taxes and profits in China, approximately 7.6% of the government's total revenue [3]. It is suggested that close to 90% of lung cancer mortality is due to cigarette smoking or inhalation of second-hand smoke. The link is even stronger in elderly smokers. The chance of dying from lung cancer in male smokers is eight- to 20-fold that of non-smokers. The risk of lung cancer is directly proportionate to the amount of cigarettes consumed. The incidence of lung cancer is 75 in 10,000 people who smoke less than 14 cigarettes daily, but this number increases dramatically to 227 in smokers who smoke more than 25 cigarettes a day. Similarly, the duration of cigarette use also proportionately correlates with the risk of developing lung cancer. It is an extremely difficult task to control the use of cigarettes and to prevent lung cancer in China. China has been hit the hardest by tobacco industries with more than one million people dying annually of tobacco-related health problems. If this trend continues, it is estimated that over two million people by 2030 and possibly three million people by mid-century may die of tobacco-related illnesses. By eliminating smoking, it is estimated that the total mortality could be reduced by 10.0% among men and by 3.5% among women in China [3].

Air pollution: China is facing a serious environmental crisis due to widespread pollution. Air pollution contributes to the high incidence of respiratory ailments and lung cancers. The main sources for air pollution in China are industrial exhaust and dust, automobile exhaust, coal burning for energy, etc., which are present mostly in major cities [4, 5].

Environmental and occupational exposures: Occupational exposure to asbestos, silicon, chromium, nickel and their by-products are known to cause lung cancer. Workers who work at charcoal mills and in the mining industries also have a higher risk of lung cancer due to chronic exposure to the dust which contains silicon, arsenic and radon gas.

Genetic susceptibility

### Liver Cancer-Hepatocellular Carcinoma (HCC)

The risk factors for liver cancer in China:

Hepatitis B viral (HBV) infection: Hepatitis B is an endemic in China. A national survey in 2002 showed a 9% rate of hepatitis B surface antigen (HbsAg) in the general population [6]. Around 130 million people in China are carriers of hepatitis B virus (HBV) (almost a third of the people infected with HBV worldwide); 30 million people in China are chronically infected [7]. Every year, 300,000 people die from HBV-related diseases in China, including 180,000 patients with HCC [8]. The incidence of hepatitis B is still increasing from 21.9 in 100,000 people in 1990 to 53.3 in 100,000 in 2003 [9]. That increase has occurred despite a vaccination program for newborn babies since the 1990s. By 2006, China has successfully immunised 11.1 million children living in the country's poorest provinces against hepatitis B according to the Chinese health ministry, and the Global Alliance for Vaccines and Immunization (GAVI).

Cirrhosis: During a five-year period, 10–20% of patients with chronic hepatitis developed cirrhosis, and 20–23% of the cases with compensated cirrhosis progressed to decompensated cirrhosis. Six to 15% of the people with cirrhosis and chronic hepatitis progressed to HCC. The five-year survival for compensated cirrhosis is 55%, for decompensated cirrhosis is 14%, and for HCC is less than 5%. Approximately 85% of liver cancer patients also suffer from cirrhosis, typically nodular type. In recent years, hepatitis C related cirrhosis has become as prevalent as that of hepatitis B associated cirrhosis.

Afflatoxins: Afflatoxins B1 (AFB1) is a well-known carcinogen for liver cancer. These toxins are made by fungus that contaminates peanuts and corn. Once ingested, these toxins can cause liver cancer directly or enhance the risk from hepatitis B infection.

Contamination of drinking water: A strong association exists between the incidence of liver cancer and the consumption of polluted water in rural areas of China.

Alcohol consumption: Excessive drinking of alcohol remains a risk factor for HCC. Some studies have also shown an increased risk of HCC in those that use alcohol and tobacco due to an additive effect.

Parasite infection: A parasitic worm, commonly known as a fluke, infests rivers in rural parts of China. Flukes can stimulate proliferation in the epithelium of the biliary duct during feeding and migration, which in turn can induce cholangiobiliary carcinoma.

Genetic susceptibility and hereditary

### Gastric cancer and its risk factors

Dietary: Unhealthy diets and food preparations can introduce carcinogens for gastric cancer. The most well-known substances are nitrates and nitric acid, nitrous acid, complex aromatic hydrocarbon and its byproducts. These may contaminate the food or can be produced during processing of the food, and has been shown to increase the risk for stomach cancer as demonstrated in some regions in China where smoked fish and meat are frequently consumed. There is some evidence to suggest a correlation of stomach cancer with diets high in sodium. The deficiency in certain nutrients such as animal proteins, vitamins and antioxidants may also play a role in the development of gastric cancer.

Infections: *Helicobacter pylori* is a well-known cause for certain stomach cancers.

Genetics: A positive family history also increases the risk for gastric cancer.

### Oesophageal cancer and its risk factors:

Chemicals and environmental factors, including N-nitrites, mould-infected foods, toxins produced by fungi, a deficiency in minerals, infectious processes such as fungal and human papilloma virus (HPV), genetic susceptibility and an unhealthy lifestyle including the use of tobacco and alcohol all increase the risk for oesophageal cancer. The use of pesticides, chemical fertilisers, and the consumption of contaminated water has been linked to the development of oesophageal cancer.

### Colorectal cancer and its common risk factors:

Dietary: There is a strong association between colon cancer and nutritional, hereditary and environmental factors. The effect of carcinogens and familial hyper-susceptibility can induce gene mutation leading to cancer development. With the marked improvement of living standards in China, the Chinese are no longer faced with starvation; instead they have an abundance of choices for nutrition. There is a significant change in the diet in China; high calories, high fat, high protein and less fibre now comprise the typical Chinese diet. The unhealthy diet and more westernised lifestyle may have contributed to the increase of colorectal cancer in China.

Genetics: Hereditary factor plays a very important role in colorectal cancer. It is estimated that 5 to 20% of colon cancers may be due to hereditary conditions such as hereditary non-polyposis colon cancer (HNPCC), and familial adenomatous polyposis (FAP).

Certain gastrointestinal ailments can also increase the risk for colon cancer such as ulcerative colitis, Crohn’s disease, and adenomatous polyposis, all of which have increased in China.

## THE MANAGEMENT OF LUNG AND BREAST CANCERS IN CHINA

### The strategies in the prevention and treatment of lung cancer

1. Prevention: The single most effective measure to reduce lung cancer in China is smoking cessation and the elimination of all tobacco products in China. This has proven to be extremely difficult to accomplish due to various historical, cultural, social and economic influences. A concerted effort has not been established by the government to ban smoking.
2. Early diagnosis and intervention: Early diagnosis and intervention can improve the treatment outcome for lung cancer. Unfortunately, effective and practical measures to screen for lung cancer in China are not available. (Or not established?)
3. Treatments:
  - Minimum invasive treatments such as video-assisted thoracoscopy and thoracotomy surgery (VATS) and radiofrequency ablation (RFA) can be used in early stages or highly-selected patients.
  - Comprehensive multi-modality therapy with a combination of local and systemic therapy can maximise the treatment outcome for more advanced stage lung cancers. Systemic therapy includes chemotherapy, targeted therapy and rehabilitation.
  - Effective health care policy: It is necessary to increase governmental funding for the prevention, diagnosis and treatment of lung cancer. The main focus should be curtailing the use of tobacco in China.

### The practice guidelines of lung cancer

The main treatment modalities for lung cancer are surgery, radiation therapy and chemotherapy. More recently bio-molecular targeted therapy has been incorporated into the comprehensive strategies of lung cancer management. In China, there are 58 Positron Emission Tomography-Computed Tomography (PET-CT) scanners which have played an important role in improving lung cancer staging and radiation treatment planning.

Surgery remains the main treatment modality for early stage (I-IIb) non-small cell lung cancer (NSCLC). Over the last decade there have been significant advancements in minimum invasive surgery in China. This reduces the risk of surgery in elderly patients and those with significant co-morbidities such as chronic obstructive pulmonary disease (COPD). Adjuvant radiation therapy and/or chemotherapy are used in selected patients. For advanced stage NSCLC, chemotherapy or supportive care are the treatments of choice (table 3).

**Table 3:** Chemotherapy regimens for non-small cell lung cancer NSCLC.

**First line**	
**NP-vinorelbine-vavelbin & platinum**	
Platinum	80 mg/m^2^ IV gtt on day 1
Vinorelbine	25 mg/m^2^ IV gtt on day 1,8
Repeat every 3 weeks	
**GP-gemcitabine & platinum**	
Platinum	75 mg/m^2^ IV gtt on day 2
Gemcitabine	1250 mg/m^2^ IV gtt on day 1,8
Repeat every 3 weeks	
**EP-etoposide (VP-16)& platinum**	
Platinum	75 mg/m^2^ IV gtt on day 1
VP-16	120 mg/m^2^ IV gtt on day 1,2,3
Repeat every 3 weeks	
**PT-platinum & Paclitaexel**	
Paclitaxel	175 mg/m^2^ IV gtt day 1
Platinum	75 mg/m^2^ IV gtt on day 1
Repeat every 3 weeks	
**PD-platinum and docetaxel**	
Platinum	75 mg/m^2^ IV gtt on day 1
Docetaxel	75 mg/m^2^ IV gtt on day 2
Repeat every 3 weeks	
**CPT-11/DDP**	
Platinum	60 mg/m^2^ IV gtt on day 1
CPT-11	60 mg/m^2^ IV gtt on day 1,8,15
Repeat every 3 weeks	
**PC-paclitaxel & carboplatin (CBP)**	
CBP	AUC5 IV gtt on day 1
Paclitaxel	175 mg/m^2^ IV gtt on day 1
Repeat every 3 weeks	
**Second line chemotherapy**	
Docetaxel	75 mg/m^2^ IV gtt on day 1
Repeat every 3 weeks	
Pemetrexed	500 mg/m^2^ IV gtt on day 1
Repeat every 3 weeks	

### Radiation therapy for NSCLC

#### Conventional radiation therapy

The treatment volumes cover the primary with a margin of 1.5 – 2cm and ipsilateral hilar and mediastinal lymph nodes. The standard treatment has generally been 1.8 -2 Gy per fraction, one fraction per day, five days per week, for a total dose of 40 Gy to 66 Gy. The initial volume is treated to a dose of 40 Gy, and then the area of residual disease detected on a repeat CT scan will be boosted to a total dose of 66 Gy.

#### Intensity modulated radiation therapy (IMRT)

The treatment volume is defined according to ICRU 50. Gross target volume (GTV) is defined as the known volume of the primary tumour and its regional nodal metastases clinically and radiologically. GTV will be contoured on the lung window of the CT scan. Clinical target volume (CTV) is defined as GTV plus 1cm margin to include surrounding subclinical disease and the area at risk for microscopic involvement. The radiologists, radiation oncologists and physicists work together to define the CTV. Planning target volume (PTV) is CTV plus 1.5 cm. PTV is covered by 90% isodose line. Lung V20 ≤ 20%. Dose to oesophagus ≤ 40 Gy and to heart ≤ 30 Gy. Median dose is 64.8 Gy (60 Gy to 72 Gy), 1.8 – 2 Gy per fraction, one fraction per day and 5 fractions per week.

#### Targeted therapy

Several new bio-molecular targeted therapy agents, such as gefitinib, erlotinib and bevacizumab, have become available in China.

### Small cell lung cancer (SCLC)

The treatment of small cell lung cancer mainly involves chemotherapy. Table 4 shows the most common regimens that are used in China.

**Table 4:** Most common treatments for small cell lung cancer (SCLC) used in China.

**Chemotherapy:**	
**EP: Etoposide (VP-16) & cisplatin (CDDP)**	
CDDP	75 mg/m^2^ IV gtt on day 1
VP-16	100-120 mg/m^2^ IV gtt on day 1, 2, 3
Repeat every 3 weeks	
**Other regimens include:**	
**EC: Etoposide & carboplatin (CBP)**	
Repeat every 3 weeks	
**CDDP & CPT-11**	
Repeat every 4 weeks	
**Radiation therapy (RT):**	
Thoracic RT:	40-50 Gy/4-5 weeks
Prophylactic whole brain irradiation:	30-36 Gy/10-12 fractions/2-2.5 weeks

### Management of Breast Cancer

Table 5 shows the chemotherapy regimens for breast cancer.

**Table 5:** Chemotherapy regimens for breast cancer.

**CMF**	
Cyclophosphomide (CTX)	100 mg/m^2^, po, day 1-14
Methotrexate (MTX)	40 mg/m^2^, IV gtt, on day 1, 8
5-Fu	600 mg/m^2^, IV gtt, on day 1, 8
Every 28 days/cycle	
**FAC**	
5-Fu	500 mg/m^2^, IV gtt, on day 1, 8
Adriamycin (ADM)	50 mg/m^2^, IV on day 1
CTX	500 mg/m^2^ IV on day 1
Every 21 days/cycle	
**AC**	
ADM	60 mg/m^2^, IV, on day 1
CTX	600 mg/m^2^, IV, on day 1
Every 21 days/cycle	
**TAC**	
TXT	75 mg/m^2^, IV gtt, on day 1
ADM	50 mg/m^2^, IV gtt, on day 1
CTX	500 mg/m^2^, IV, on day 1
Every 21 days/cycle	
**AC->T**	
ADM	60 mg/m^2^, IV, on day 1
CTX	600 mg/m^2^, IV, on day 1
Every 21 days/cycle x 4 cycles	
Paclitaxel	175 mg/m^2^ IV gtt, on day 1
Every 21 days/cycle x 4 cycles	
**FEC**	
5-Fu	400 mg/m^2^, IV gtt, on day 1, 8
EPI	50 mg/m^2^, IV, on day 1, 8
CTX	500 mg/m^2^, IV, on day 1, 8
Every 28 days/cycle	

#### Post-mastectomy radiation therapy

The radiation target volume post-radical or modified radical mastectomy includes chest wall and regional lymph nodes. The indication of radiation and the identification of target volume are dictated by the stage of the primary tumour and regional lymph nodes.

Chest wall: The upper border is the thoracic entrance or to match the lower border of the supraclavicular field (half-beam block). The lower border is 1-2 cm inferior to the breast tissue. The medial border is placed just medial to the inner breast tissue or the midline of sternum. The lateral border is the midline of axilla. The chest wall post-modified radical mastectomy is irradiated using 4 -6 MV x-ray via standard tangent fields with wedges and bolus. The chest wall post-radical mastectomy is irradiated en-face using electrons. The energy of the electrons is selected according to the thickness of the chest wall. Bolus is used to increase the skin dose to ≥ 85%. The total dose will be 45 Gy to 50 Gy.

Supraclavicular lymphatic drainage: The upper border extends laterally across the neck and the trapezius to the acromial process. The lower border is at the first or second intercostal space. The medial border is the midline of the sternum or 1 cm beyond. The lateral border is the anterior line of axilla. The dose covers a depth of 3-4 cm. The mixed beams of photons (^60^Co or 4-6 MV x-ray) and electrons (12 MeV) are used to deliver 45 – 50 Gy in conventional fractionation.

Internal mammary chain: The upper border is at the medial head of clavicle or to match the lower border of the supraclavicular field. The lower border is in the fourth intercostal space. For a primary tumour located at the inner lower quadrant, the lower border is at the sixth intercostal space. The medial border is the midline of sternum or 1 cm beyond. The lateral border is 5 cm from the midline of sternum to the ipsilateral chest wall. The dose covers a depth of 3-5 cm. Electrons with appropriate energy are used to deliver 45 – 50 Gy in conventional fractionation.

Axillary lymphatic drainage: The upper border is at the clavicle. The lower border is at the second intercostal space. The medial border is 1-1.5 cm lateral to the chest wall. The lateral border is at the posterior line of the axilla. The 4-6 MV x-ray is used to deliver 45 – 50 Gy in conventional fractionation.

#### Post-lumpectomy radiation therapy

Tangential fields and boost fields: The borders of the tangential fields are usually defined as follows: medial border is midline of the sternum (or 3 cm beyond midline to include the internal mammary chain); lateral border is mid-axillary; inferior border is 1-2 cm below the breast; superior border is the thoracic entrance or to match the inferior border of the supraclavicular field. The total dose is 45-50 Gy at 1.8 -2 Gy per fraction, 5 fractions a week.

Accelerated partial breast irradiation: There are many ways to deliver partial breast irradiation either with brachytherapy including; interstitial brachytherapy, intracavitary brachytherapy or an external beam radiation therapy system such as a 3D Conformal radiotherapy or IMRT and intraoperative radiotherapy (IORT).

#### Hormone therapy for breast cancer

Hormone therapy is commonly used for patients with breast cancer that stain positive for oestrogen receptor (ER) and progestron receptors (PR). Targeted therapy such as trastuzumab is used for Her2 receptor positive patients respectively.

## CURRENT STATUS OF RADIATION ONCOLOGY IN CHINA

### Radiotherapy Equipment in China

There is a significant shortage in radiotherapy equipment to meet the increasing needs in China. As of September 2006, there were 952 radiation therapy centers that were equipped with 918 linear accelerators, 472 ^60^Co machines, 146 ortho-voltage x-ray machines, 827 fluoroscoopy simulators, and 400 brachytherapy units including 21 ^252^Cf neutron remote afterloading brachytherapy units. There were 851 treatment planning systems and 467 ion-chambers [10]. In comparison to the 1997 survey, there has been a 321% increase in linear accelerators and 481% increase in treatment planning systems. As of 2006, there were 214 CT simulators, 149 Gamma Knives (74 for head only, 75 for both head and body), and 467 X-Knife machines. Despite the significant growth in radiation oncology equipment, there is less than 1 (0.7) accelerator per million people. Even considering both ^60^Co machines and linear accelerators combined, there are only 1.06 machines per one million population, which is short of meeting the minimum requirements of the World Health Organization of two linear accelerators per million population. The radiotherapy equipment is also unevenly distributed throughout the country. In the major cities or provinces such as Beijing, Shanghai, and Shandong, there are at least two machines per million people, whereas in more remote regions of China such as deep inland and the western part of the country, there is limited accessibility to radiotherapy equipment.

### Radiation Technique

The majority of radiation centers in China are equipped and capable of performing basic three dimensional conformal radiotherapy (3D-CRT) as reported by the 2006 survey [10]. Among 952 registered radiation centers in China, 579 centres (60%) were practising three-dimensional conformal radiotherapy, but only 115 (12%) were delivering intensity-modulated radiotherapy (IMRT). Approximately 8% of centres were capable of doing stereotactic radiosurgery [10].

### Radiation Oncologists and Radiation Physicists in China

There are 18,992 healthcare workers that are involved in providing radiation therapy in China. Among them, 5,247 are radiation oncologists (including 2,110 residents), 4,559 radiation therapists, 6,864 nurses, 1,181 medical physicists, and 1,141 are maintenance engineers. Clearly there is a severe shortage in medical physicists.

### Radiation Therapy Funding in China

In China, there is a significant lack of funding and reimbursement to maintain costly radiotherapy equipment and meet the demands. A national health insurance system in China has not been developed. The reimbursement rates for cancer treatment are set by the local governments and vary widely due to the differences in the financial situations of each district. For example, in City of Qingdao the municipal healthcare agency reimburses 80% for IMRT treatment. However, at the same time they also set a fixed reimbursement rate of 250 Chinese yuan (equivalent to approximately $40) for each IMRT beam, 80% of which will be reimbursed. The government will only pay 70% of the radiation treatment planning cost. The rest of the expense has to be paid by the patients. For 3D-CRT the local government has set the maximum reimbursement rate of 1,100 yuan (equivalent to approximately $150). Therefore, a significant portion of the expense will have to be paid by the patients or they have to accept substandard or no treatment at all. The same situation also applies to chemotherapy treatments. The government will pay the full amount of money, but only for the basic drug regimen. However, the newer or more expensive chemotherapy drugs (which may be more effective) will get little or no reimbursement. Furthermore, 60% of the Chinese population have no medical insurance or coverage at all. Unfortunately for those patients, they either have to pay completely out-of-pocket (which very few can afford), or settle for less service, which oftentimes results in inadequate healthcare. As most people either have no healthcare insurance or have an insurance coverage with low reimbursement rates, it is very difficult, if not impossible, for them to receive comprehensive or long-term care. There is also a profound disparity of healthcare within China. In eastern regions of China, especially large coastal cities, medical care is more advanced and relatively accessible than that in remote areas such as many western provinces.

## UNIQUE CANCER THERAPY IN CHINA

China has a long history of using its traditional Chinese medicine for various ailments, including malignancies. Clinical and experimental research have shown that Chinese medicine may have positive effects on cancer and cancer therapy. The potential benefits of traditional Chinese medicine are believed to be as follows:

1. Reducing the toxic side-effects of chemotherapy and radiation therapy.
2. Improving human immune systems and its functions.
3. Improving haematopoiesis in bone marrow.
4. Improving endocrine function and body metabolism
5. Re-establishing homeostasis to promote the human body’s recovery.
6. Enhancing the treatment effects which may lead to a prolonged survival.

### Qigong

*Qigong* (an ancient Chinese exercise system of deep breathing) is also believed to improve a patient’s overall well-being by adjusting each organ’s function. It further improves the patient’s immune system and boosts one’s confidence. *Qigong* may also perk up a patient’s emotional and psychological state of health leading to a better tolerance of cancer therapy and a faster recovery.

Incorporating Chinese medicine into a comprehensive cancer treatment program may enhance the overall treatment outcomes. In recent years, the Chinese government has approved the use of some Chinese herbal remedies that may improve the immune function of cancer patients and also may have additive effects to conventional cancer therapy.

### Chinese Herbal Medicine

Chinese herbal medicine has existed and been practised in cancer therapy in China for ages. It is administered based on the underlying physio-pathologic processes of the malignancies, including the type and stage of the malignancy, the patients’ overall state of health and nutritional status. Chinese medicine remedies also take other treatment modalities into consideration such as the side-effects from the treatment (e.g. chemotherapy or radiation) and clinical symptoms. It is believed that Chinese herbal medicine may lessen the patient’s clinical symptoms, decrease toxic side-effects from conventional western approaches and result in favourable therapeutic ratios. The use of Chinese herbal medicine is a very complicated process that contains the art and science of medicine as well as human experiences, wisdom, and knowledge that have been accumulated and passed on for generations in China. The combination of conventional western medicine and traditional Chinese medicine may be complementary to each other and may ultimately improve the cancer care in China.

### ^252^Cf neutron remote afterloading brachytherapy

China has a relatively large number of remote afterloading neutron brachytherapy units using Californium-252 (^252^Cf) nuclide which is a source of gamma neutron radiation. ^252^Cf generated by nuclear reactions produces fast neutrons which offer high-LET radiation and potentially a more effective cell kill effect than photons. The indication and technique of ^252^Cf are similar to those that are used with ^192^Ir for treating intra-cavitary/intra-luminal or body surface tumours such as oesophageal and cervical cancer. ^252^Cf is, at least theoretically, equal to or more effective than photons generated by ^192^Ir. Because of its high-LET radiation ^252^C may offer more favourable treatment outcomes for tumours that have large hypoxic cell population or tumours that did not respond to conventional photon radiation. For difficult or recurrent tumours with hypoxic cells, ^252^Cf neutron therapy may also produce a faster tumour response compared to that of photon beam.

### Dendritic Cell Immunotherapy

China has a very active program in basic and clinical research on dendritic cell immunotherapy. A new and more efficient way to culture LAK cells was developed in China. This allowed a more efficient way to produce LAK cells for possible clinical use. This new approach was used in a clinical trial using cultured CD3AK cells in 1999. This therapy has the following advantages: 1) it can be used for a wide range of malignancies, 2) it has significant effects on both primary tumours and metastases, 3) it may be more effective than conventional chemotherapy in adjuvant setting, and 4) the side effects are mild (only low-grade fevers). Based on early clinical evidence, this new modality appeared to be more efficacious when combined with chemotherapy to treat advanced stage cancers. The tumour response rate has been reported to be as high as 60%. It was the first time that the DC + CIK was combined with chemotherapy to treat late stage lung cancer and combined with neutron knife to treat colon cancer in clinical trials conducted in China. Further research and larger clinical trials are being done in this area.

## THE HEALTHCARE POLICIES

China is experiencing a rapid growth in the aging population and cancer incidence. In the past, the government health agencies were focused more on cancer treatment than its prevention. Recently the Chinese government has started to attribute more attention towards cancer prevention and cancer therapy rehabilitation. Fortunately, the basic needs for surgical oncology equipment have been met and many radiation therapy facilities have been developed in the last 20 years. More and more linear accelerators and ^60^Co machines have been installed nationwide. But it is far from meeting the basic needs in China. Chemotherapy is also advancing and has adopted the international standards in many parts of China. The concept and practice of terminal care have also been introduced into more families, healthcare institutions and social welfare organisations in today’s practice. The government has also allowed more use of narcotics for pain control for terminal cancer patients. As an example, the city of Qingdao was one of the first cities in the nation to start an education centre for cancer patients which is supported by the local government. The centre is open to the public. It provides guidance and assistance on a wide range of issues in cancer care and cancer therapy rehabilitation. The centre also provides telephone hotlines for psychological counseling for cancer patients. Patient support groups meet regularly to discuss the variety of issues that they are facing, including cancer therapy, the coping process and rehabilitation. Oncologists are invited to give lectures and advice to the participating groups.

The central government has realised that the cost of cancer treatment is overwhelming and will continue to grow exponentially in the foreseeable future. The best way to curtail the cost in cancer care is cancer prevention. The current health insurance system provided by the local government does not provide funding for preventive measures, which has been a major obstacle to the implementation of preventive medicine. Since 2004, the Chinese government has made efforts to increase funding for cancer prevention and early detection which are important to reducing cancer-related deaths in China. Multidisciplinary management has become the principle of cancer treatment, and efforts to practise the standard of care have been implemented. However, there remains a large number of Chinese people who do not have health insurance or have coverage that is inadequate as the cost extends beyond their financial ability to even afford the co-pay. Therefore, cancer patients are facing heavy financial burdens which may prevent them from obtaining little, if any, cancer therapy. These problems are much more profound in the rural areas of China.

## THE FUTURE CHALLENGES

Cancer care is a complicated process that requires an enormous effort and resources from the government, healthcare workers and cancer researchers. It is estimated that 80% of cancers are preventable; therefore, it is essential to control environmental pollution and to advocate a healthy lifestyle and diet. An increase in the awareness of cancer prevention and early detection through educating the public has become paramount. It is absolutely necessary for both the central and local governments to greatly increase the funding for cancer care and to improve the distribution of the resources throughout China.
